# MiR-3976 regulates HCT-8 cell apoptosis and parasite burden by targeting *BCL2A1* in response to *Cryptosporidium parvum* infection

**DOI:** 10.1186/s13071-023-05826-w

**Published:** 2023-07-06

**Authors:** Juanfeng Li, Lulu Sun, Fujie Xie, Tianren Shao, Shanbo Wu, Xiaoying Li, Longxian Zhang, Rongjun Wang

**Affiliations:** College of Veterinary Medicine, Henan Agricultural University, Zhengzhou, 450046 China

**Keywords:** *Cryptosporidium parvum*, miR-3976, *BCL2A1*, Apoptosis, Parasite burden

## Abstract

**Background:**

*Cryptosporidium* is second only to rotavirus as a cause of moderate-to-severe diarrhea in young children. There are currently no fully effective drug treatments or vaccines for cryptosporidiosis. MicroRNAs (miRNAs) are involved in regulating the innate immune response to *Cryptosporidium parvum* infection. In this study, we investigated the role and mechanism of miR-3976 in regulating HCT-8 cell apoptosis induced by *C. parvum* infection.

**Methods:**

Expression levels of miR-3976 and *C. parvum* burden were estimated using real-time quantitative polymerase chain reaction (RT-qPCR) and cell apoptosis was detected by flow cytometry. The interaction between miR-3976 and B-cell lymphoma 2-related protein A1 (*BCL2A1*) was studied by luciferase reporter assay, RT-qPCR, and western blotting.

**Results:**

Expression levels of miR-3976 were decreased at 8 and 12 h post-infection (hpi) but increased at 24 and 48 hpi. Upregulation of miR-3976 promoted cell apoptosis and inhibited the parasite burden in HCT-8 cells after *C. parvum* infection. Luciferase reporter assay indicated that *BCL2A1* was a target gene of miR-3976. Co-transfection with miR-3976 and a *BCL2A1* overexpression vector revealed that miR-3976 targeted *BCL2A1* and suppressed cell apoptosis and promoted the parasite burden in HCT-8 cells.

**Conclusions:**

The present data indicated that miR-3976 regulated cell apoptosis and parasite burden in HCT-8 cells by targeting *BCL2A1* following *C. parvum* infection. Future study should determine the role of miR-3976 in hosts’ anti-*C. parvum* immunity in vivo.

**Graphical Abstract:**

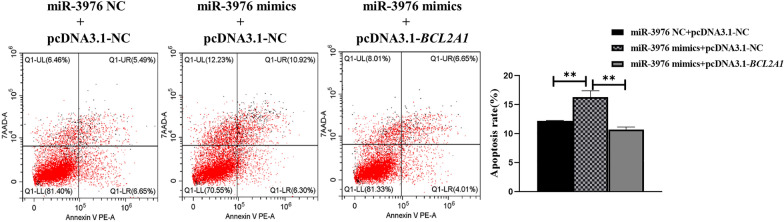

**Supplementary Information:**

The online version contains supplementary material available at 10.1186/s13071-023-05826-w.

## Background

*Cryptosporidium parvum* is a zoonotic apicomplexan protozoon and a causal pathogen of cryptosporidiosis [1]. The development of cryptosporidiosis depends largely on the immune status of the infected individual; self-limited diarrhea is the main symptom in immunocompetent individuals, but infection can lead to persistent diarrhea and death in immunocompromised individuals, especially AIDS patients [2–4]. *Cryptosporidium* is the second-leading cause of diarrhea-associated mortality in children under 5 years of age, and was responsible for 604,000 deaths in 2015 [5, 6]. *Cryptosporidium parvum* has been recognized as one of the most common and dangerous opportunistic parasites causing diarrhea, and a significant association has been demonstrated between *C. parvum* and malnutrition, including growth failure and wasting, in children in developing countries [7–10]. There are currently no effective drug treatments or vaccines for cryptosporidiosis. Nitazoxanide is the only approved drug for cryptosporidiosis, but its efficacy is limited in immunocompetent individuals and it is not effective in immunocompromised individuals, especially HIV/AIDS patients [11]. There is thus an urgent need to develop new drugs and/or vaccines to treat cryptosporidiosis. However, the pathogenic mechanism of cryptosporidiosis remains largely unknown, and this lack of information limits the availability of effective treatment and prevention strategies.

MicroRNAs (miRNAs) are small non-coding RNAs that regulate gene expression at the post-transcriptional level by pairing to the 3′-untranslated region (3′-UTR) of target messenger RNAs (mRNAs), either perfectly or imperfectly [12]. This interaction regulates a series of cellular processes including cell proliferation, apoptosis, and differentiation [13, 14]. Because *C. parvum* lacks the key components required for small RNA-mediated post-transcriptional gene silencing, it is an ideal model for investigating host miRNA-mediated defenses against infection in epithelial cells [15]. Increasing evidence has demonstrated that miRNAs may modulate the host epithelial immune responses following *C. parvum* infection [16]. For example, *C. parvum* infection induced the expression of *let-7i* and miR-513, which contributed to cholangiocyte immune responses against *C. parvum* infection in vitro [17–19]. Some evidence also suggested that *C. parvum* utilized host miRNAs to escape the host defense and promote its own survival [19, 20]; for example, transcriptional activation of miR-27b targeted KH-type splicing regulatory protein (*KSRP*) to regulate the burden of *C. parvum* infection [20]. Studying the mechanism by which miRNAs regulate epithelial immunity against *C. parvum* infection will further our understanding of the interaction between *C. parvum* and its host, and aid the screening of potential drug targets.

Apoptosis is considered to be an important defense mechanism against *Cryptosporidium* infection during innate immunity [21]. *Cryptosporidium parvum*-induced apoptosis in infected and uninfected biliary epithelial cells via a factor-associated suicide (Fas)/Fas ligand (FasL)-dependent mechanism, which disturbs parasite propagation and facilitates clearance [22]. However, although studies on miRNA expression profiles have demonstrated the inductive potential of *C. parvum* with respect to apoptosis [23], little is known about the regulation of host cell apoptosis by miRNAs following *Cryptosporidium* infection. We previously examined the miRNA expression profiles of HCT-8 cells infected with *C. parvum* and found that miR-3976, miR-942-5p, miR-181d, miR-18b-3p, miR-34b-5p, and miR-3591-3p regulated apoptosis during the early phase of infection [23]. The present study aimed to clarify the mechanism by which miR-3976 regulates HCT-8 cell apoptosis following *C. parvum* infection.

## Methods

### Cell culture and *C. parvum* infection model

The human ileocecal adenocarcinoma (HCT-8) cells (American Type Culture Collection, Manassas, VA, USA) were cultured in Dulbecco’s modified Eagle’s medium supplemented with 10% fetal bovine serum, 4 mmol/l l-glutamine, 100 U/ml penicillin, and 100 U/ml streptomycin at 37 °C in 5% CO_2_. Neonatal calves were purchased from Ruiya Animal Husbandry Co., Ltd. (Zhengzhou, China). *Cryptosporidium parvum* subtype IIdA19G1 oocysts were passaged and maintained in neonatal calves and stored in 2.5% K_2_Cr_2_O_7_ solution at 4 °C after purification. The oocysts used in the experiment have been passaged for about 20 generations. Oocysts were excysted in 0.25% trypsin and 0.75% sodium taurocholate for 1 h, with mixing every 5 min, followed by incubation at room temperature for 30 min, as described previously [24], followed by washing three times in phosphate-buffered saline (PBS) and resuspension. Monolayer cells in 6- or 12-well plates were inoculated with sporozoites at a ratio of 5:1 sporozoites to cells.

### Real-time quantitative polymerase chain reaction (RT-qPCR)

HCT-8 cells were washed three times with PBS and total RNA was isolated using TRIzol reagent (Invitrogen, Waltham, MA, USA) following the kit manufacturer’s instructions, and subsequent to treatment with Recombinant DNase I (Takara, Kyoto, Japan). Extracted RNA (1 μg) was reverse transcribed into complementary DNA (cDNA) and miRNA using ReverTra Ace qPCR RT Master Mix with gDNA [genomic DNA] Remover (Toyobo, Japan) and a Mir-X miRNA First-Strand Synthesis Kit (Takara Biomedical Technology, Dalian, China) following the manufacturer’s protocols. Housekeeping genes *gapdh* (glyceraldehyde-3-phosphate dehydrogenase) and *u6* were used as baselines for cDNA and miRNA quantification. cDNA or miRNA expression were determined using SYBR^®^ Green Realtime PCR Master Mix (Toyobo). RT-qPCR was performed with melting curve analysis under the following the cycling program: initial activation at 95 °C for 30 s, followed by 40 cycles of denaturation at 95 °C for 5 s, annealing at 55 °C for 10 s, and extension at 72 °C for 15 s. Fold-change differences in gene expression were calculated using the 2^−ΔΔCt^ method to show the relative expression of each RNA. The change in gene expression in treated groups compared to the control group was shown on the Y-axis. The primer sequences used in the present study are listed in Additional file 1: Table S1.

### Cell transfection

MiR-3976 mimics, inhibitors, and negative controls (NCs) were obtained from GenePharma (Shanghai, China). The B-cell lymphoma 2-related protein A1 (*BCL2A1*) overexpression vector was constructed using the pcDNA3.1 eukaryotic expression plasmid (pcDNA3.1-*BCL2A1*); the overexpression plasmid map is presented in Additional file 4: Fig. S1. Small interfering RNAs (SiRNAs) targeting *BCL2A1* mRNAs (si-*BCL2A1*) and scrambled RNA (si-NC) were designed by Sangon Biotech (Shanghai, China). Transfection was carried out using Lipofectamine 2000 reagent (Invitrogen, Carlsbad, CA, USA), according to the manufacturer’s protocols. MiR-3976 mimics and pcDNA3.1-*BCL2A1* were co-transfected with an equal quantity. The sequences of the siRNA and miRNA mimics and inhibitors are presented in Additional file 2: Table S2.

### Flow cytometry for cell apoptosis

Cells were washed with PBS at 48 hpi, digested with ethylenediaminetetraacetic acid (EDTA)-free pancreatic enzymes for 5 min, and centrifuged at 800×*g* for 5 min at room temperature. The centrifuged supernatant was removed and the cells were washed twice with PBS. Using a PE Annexin V Apoptosis Detection Kit I (BD, Denver, CO, USA), cells were resuspended with 500 µl of precooling 1× binding buffer to achieve cell density of 10^5^–10^6^/ml, followed by the addition of 5 µl of Annexin V-PE and 5 µl of 7-aminoactinomycin D. The resuspension was incubated for 15 min in the dark at room temperature. Cell apoptosis was detected using flow cytometry (Beckman Coulter, Brea, CA, USA).

### Dual luciferase assay

The sequences of wild-type (WT) *BCL2A1* 3′-UTR (*BCL2A1*-WT) and mutant *BCL2A1* 3′-UTR (*BCL2A1*-MUT) were designed and inserted into pmirGLO vectors (Promega, Madison, WI, USA). Sequences of *BCL2A1*-WT 3′-UTR and *BCL2A1*-MUT 3′-UTR are listed in Additional file 3: Table S3 and the sequences of pmirGLO-*BCL2A1*-WT and pmirGLO-*BCL2A1*-MUT are presented in Additional file 5. Each constructed plasmid was co-transfected into HCT-8 cells with miR-3976 mimics or NC using Lipofectamine 2000 reagent (Invitrogen, Gaithersburg, MD, USA). The luciferase activity of the transfected cells was measured after 48 h using the Dual-GLO^®^ Luciferase Assay System (Promega, Madison, WI, USA). Relative luciferase activity was finally calculated by firefly luciferase value/Renilla luciferase value and normalized to the activity of the co-transfected Renilla luciferase.

### Western blotting

Total proteins were extracted from 10^7^ cells using a total protein extraction kit (Solarbio Life Sciences, Beijing, China) and the protein concentrations were determined using a Pierce BCA [bicinchoninic acid] Assay Kit (Thermo Fisher Scientific, Waltham, MA, USA) following the manufacturer’s instructions. Lysed 20 µg protein samples were separated by 10% sodium dodecyl sulfate–polyacrylamide gel electrophoresis and transferred onto polyvinylidene fluoride membranes. Following blocking with 1% bovine serum albumin, the membranes were incubated overnight at 4℃ with primary rabbit antibodies against *BCL2A1* (1:1000, Abcam, Cambridge, UK) and mouse anti-GAPDH antibodies (1:2000, ImmunoWay, Tennyson, TX, USA). The membranes were then incubated with horseradish peroxidase-conjugated secondary goat anti-rabbit and goat anti-mouse antibodies (1:5000, ImmunoWay, Tennyson, TX, USA) at room temperature for 2 h. Protein bands were visualized using an enhanced chemiluminescence (ECL) system and the gray values of the protein bands were quantified using the ImageJ software.

### Statistical analysis

Statistical analysis was performed using GraphPad Prism 8.0.2 (GraphPad Software, San Diego, CA, USA). Differences between the two groups were analyzed using unpaired *t*-tests with Bonferroni correction and differences among multiple groups were analyzed using nonparametric one-way analysis of variance (ANOVA). All data were expressed as mean ± standard deviation (SD). All experiments were performed using at least three biological replicates. For all analyses, *P* < 0.05 was considered significant.

## Results

### Expression of miR-3976 was induced by *C. parvum* infection in HCT-8 cells

**Fig. 1:**
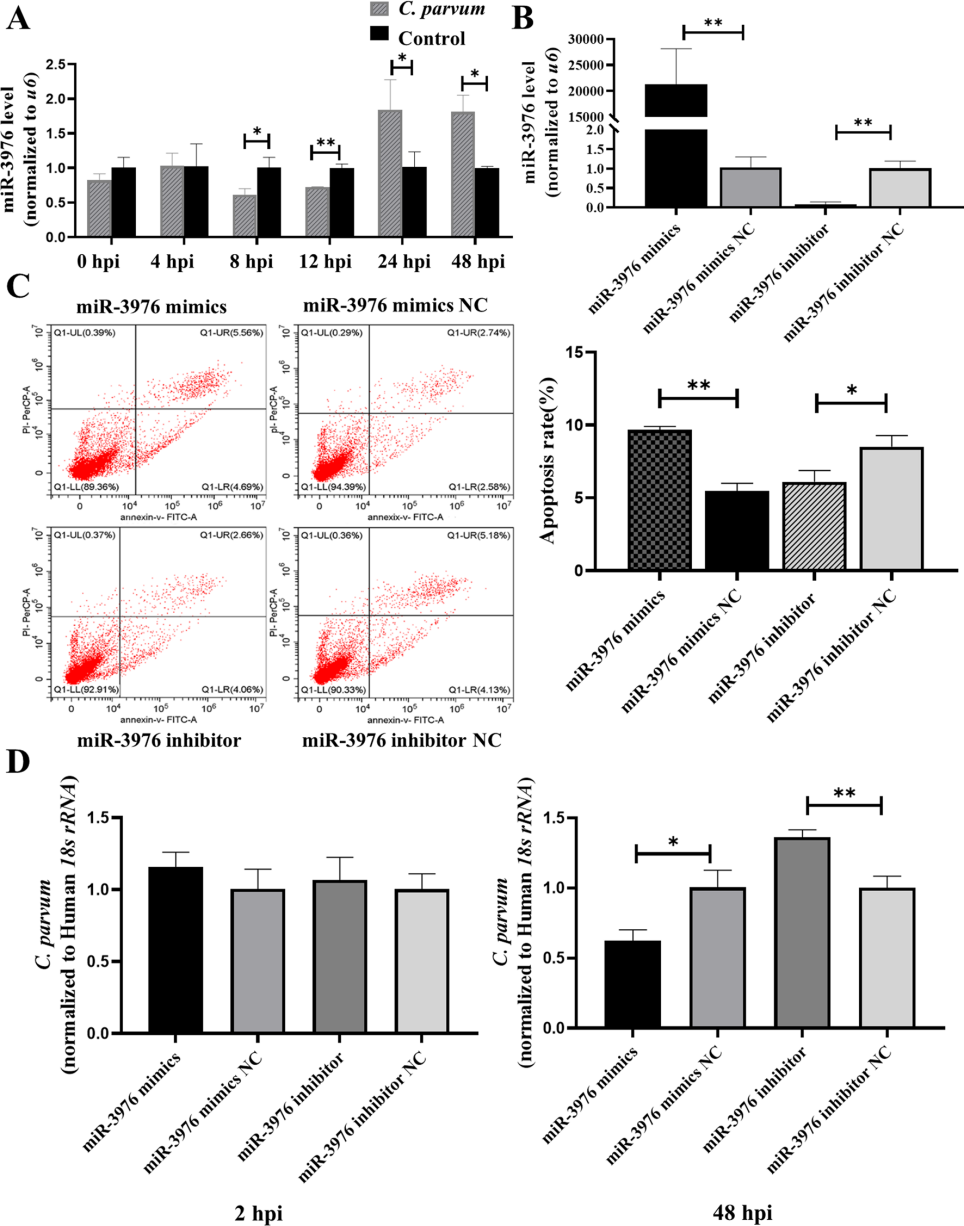
MiR-3976 regulation of cell apoptosis and parasite burden in HCT-8 cells after *C. parvum* infection. **a** Expression levels of miR-3976 in HCT-8 cells at different time points after *C. parvum* infection by RT-qPCR. **b** Expression efficiency of miR-3976 in HCT-8 cells transfected with miR-3976 mimics and miR-3976 inhibitor for 24 h by RT-qPCR. **c** Effect of miR-3976 on apoptosis of cells infected with *C. parvum*. Cells were transfected with miR-3976 mimics and miR-3976 inhibitor for 24 h and then exposed to equal numbers of *C. parvum* sporozoites for 24 h. Cell apoptosis was then detected by flow cytometry. **d** Effect of miR-3976 on parasite burden in HCT-8 cells. Cells were transfected with miR-3976 mimics and miR-3976 inhibitor for 24 h and then exposed to equal numbers of *C. parvum* sporozoites for 2 h to determine the initial cellular invasion of *C. parvum* by RT-qPCR. Simultaneously, infected cells were cultured for 48 h to evaluate the parasite burden after initial cell invasion. All data presented as combined mean ± SD of three independent experiments. Different groups were compared by *t*-tests. *P < 0.05, **P < 0.01, ***P < 0.001

A previous study of the miRNA expression profile in HCT-8 cells suggested that miR-3976 expression was decreased at 12 h post-infection (hpi) [23]. RT-qPCR results confirmed that miR-3976 expression levels were significantly decreased at 8 and 12 hpi and increased at 24 and 48 hpi compared with the control group of uninfected cells (Fig. 1a).

### MiR-3976 regulated cell apoptosis and parasite burden in HCT-8 cells after *C. parvum* infection

Functional analysis suggested that miR-3976 was involved in the regulation of apoptosis. To verify the role of miR-3976 in regulating cell apoptosis following *C. parvum* infection, we upregulated or downregulated its expression in HCT-8 cells by transfection with miR-3976 mimics or miR-3976 inhibitor, respectively (Fig. 1b), and then detected the cell apoptosis rate after infection. Flow cytometry revealed that the apoptosis rate was significantly increased in HCT-8 cells transfected with miR-3976 mimics compared with the NC group, and apoptosis was significantly decreased in the miR-3976 inhibitor group (Fig. 1c), suggesting that miR-3976 promoted HCT-8 cell apoptosis after *C. parvum* infection.

We also investigated the functional role of miR-3976 in regulating the parasite burden by RT-qPCR. The parasite burden was detected in the different groups of cells at 2 hpi, as a model for testing parasite attachment and cellular invasion, and at 48 hpi to assess parasite propagation/survival in cells [19, 20, 25]. There was no difference among the groups at 2 hpi. However, the parasite burden was increased at 48 hpi in cells transfected with miR-3976 mimics compared with the miR-3976 mimic NC group, while the opposite result was found in cells transfected with miR-3976 inhibitor (Fig. 1d). These results indicated that miR-3976 did not affect the initial parasite cellular invasion, but did inhibit the *C. parvum* burden in HCT-8 cells.

### *BCL2A1* was a target gene of miR-3976

**Fig. 2:**
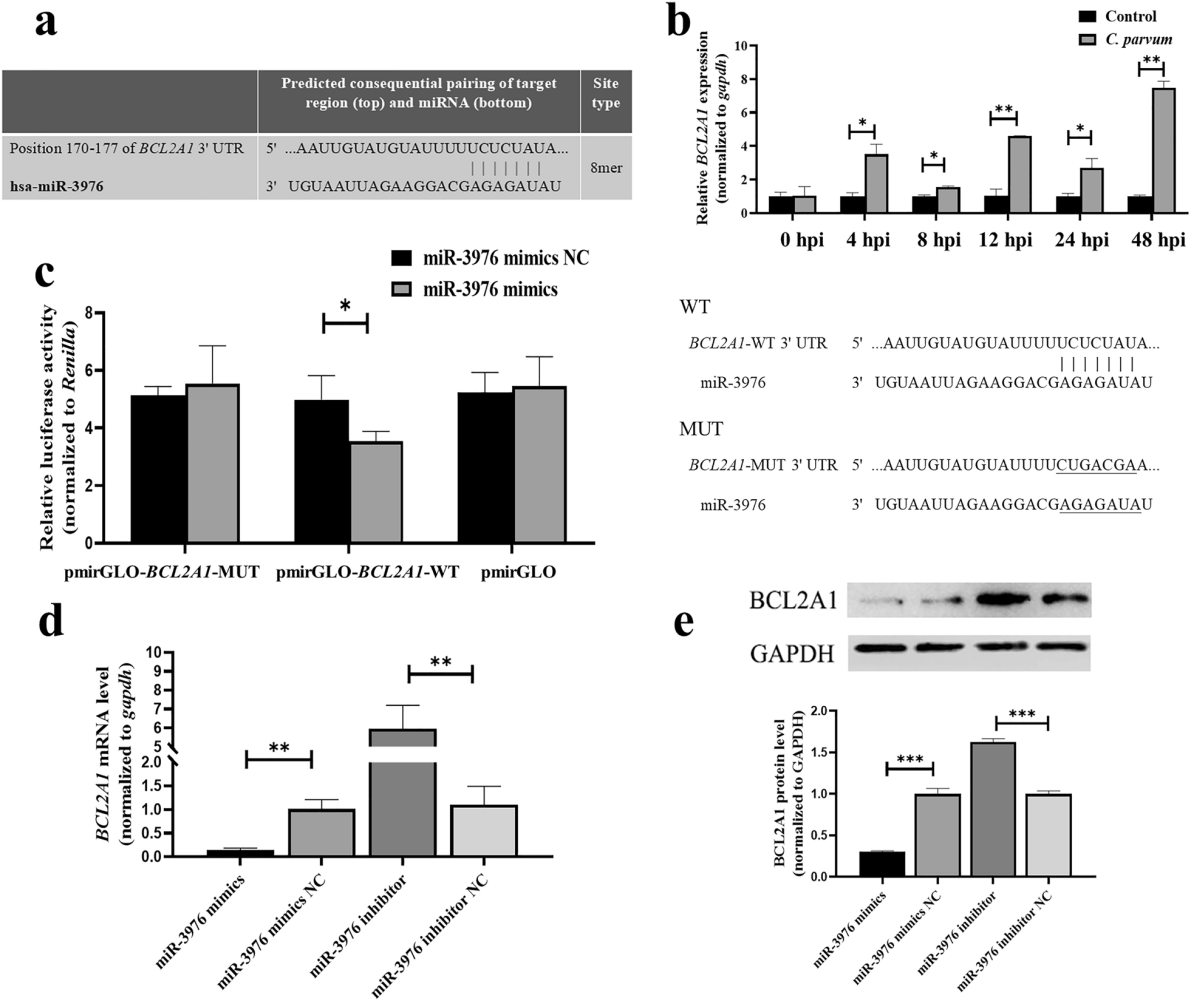
MiR-3976 regulates the expression of *BCL2A1*. **a** Binding sites of miR-3976 to *BCL2A1* predicted by TargetScan. **b** Relative *BCL2A1* expression levels in HCT-8 cells at different time points after *C. parvum* infection by RT-qPCR. **c** Binding of miR-3976 to *BCL2A1* detected by dual-luciferase activity in HCT-8 cells co-transfected with miR-3976 mimics or miR-3976 NC and pmirGLO-*BCL2A1*-WT or pmirGLO-*BCL2A1*-MUT for 48 h. The left represents the luciferase reporter assay in HCT-8 cells co-transfected with miR-3976 and *BCL2A1*, and the right represents sequences of *BCL2A1*-WT and *BCL2A1*-MUT. **d** and **e** Cells were transfected with miR-3976 mimics and miR-3976 inhibitor for 24 h and then exposed to equal numbers of *C. parvum* sporozoites for 24 h. *BCL2A1* mRNA and protein expression levels were detected by RT-qPCR and western blotting. The densitometric levels of *BCL2A1 *protein signals and mRNA levels were quantified and expressed relative to *GAPDH*. All data presented as combined mean ± SD of three independent experiments. Different groups were compared by *t*-tests. *P < 0.05, **P < 0.01, ***P < 0.001

TargetScan and miRBase were used to predict the potential target gene of miR-3976 [26, 27]. The prediction results indicated the presence of binding sites between miR-3976 and *BCL2A1* (Fig. 2a). *BCL2A1* expression levels were upregulated at different time points during *C. parvum* infection (Fig. 2b). Luciferase reporter activity was lower in cells co-transfected with *BCL2A1*-wild-type (WT) and miR-3976 mimics compared with cells co-transfected *BCL2A1*-WT and miR-3976 mimics NC, but not *BCL2A1*-MUT and pmirGLO vectors (Fig. 2c). Furthermore, overexpression of miR-3976 reduced the mRNA and protein levels of *BCL2A1*, while the opposite result was detected in cells with knockdown of miR-3976 using an inhibitor (Fig. 2d and e). These results demonstrated that miR-3976 targeted *BCL2A1* and inhibited its expression, indicating that miR-3976 might regulate HCT-8 cell apoptosis via *BCL2A1* in response to *C. parvum* infection.

### MiR-3976 regulated cell apoptosis and parasite burden in HCT-8 cells by targeting *BCL2A1* following *C. parvum* infection

**Fig. 3:**
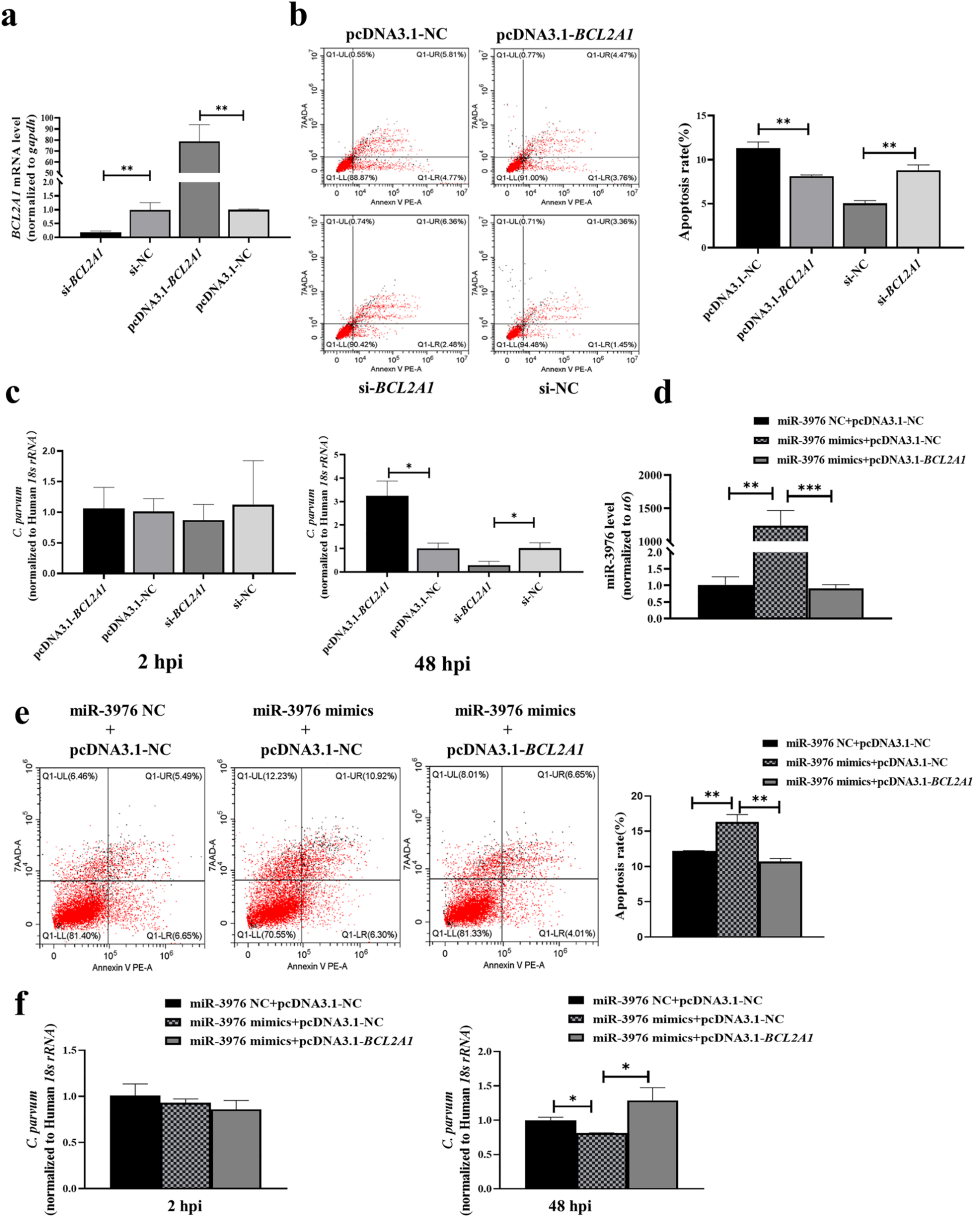
MiR-3976 directly targets *BCL2A1* to regulate cell apoptosis and parasite burden in HCT-8 cells after *C. parvum* infection. **a** Expression efficiency of *BCL2A1* in HCT-8 cells transfected with si-*BCL2A1* and pcDNA3.1-*BCL2A1* for 24 h by RT-qPCR. **b** Effect of *BCL2A1* on cell apoptosis infected with *C. parvum*. Cells were transfected with si-*BCL2A1* and pcDNA3.1-*BCL2A1* for 24 h and then exposed to equal numbers of *C. parvum* sporozoites for 24 h. Cell apoptosis was then detected by flow cytometry. **c** Regulation of parasite burden in HCT-8 cells by *BCL2A1*. Cells were transfected with si-*BCL2A1* and pcDNA3.1-*BCL2A1* for 24 h and then exposed to equal numbers of *C. parvum* sporozoites for 2 h to determine the initial cellular invasion of *C. parvum* by RT-qPCR. Simultaneously, infected cells were cultured for 48 h to evaluate the parasite burden in cells after initial cell invasion. **d** Expression efficiency of miR-3976 in HCT-8 cells co-transfected with miR-3976 mimics and pcDNA3.1-*BCL2A1* for 24 h by RT-qPCR. **e** Effect of miR-3976 targeting *BCL2A1* on apoptosis of cells infected with *C. parvum*. Cells were co-transfected with miR-3976 mimics and pcDNA3.1-*BCL2A1* for 24 h and then exposed to equal numbers of *C. parvum* sporozoites for 24 h. Cell apoptosis was detected by flow cytometry. **f** Regulation of miR-3976 targeting *BCL2A1 *on parasite burden in HCT-8 cells. Cells were co-transfected with miR-3976 mimics and pcDNA3.1-*BCL2A1* for 24 h and then exposed to equal numbers of *C. parvum* sporozoites for 2 h to determine the initial cellular invasion of *C. parvum* by RT-qPCR. Simultaneously, infected cells were cultured for 48 h to evaluate the parasite burden in cells after initial cell invasion. All data presented as combined mean ± SD of three independent experiments. Different groups were compared by *t*-tests. *P < 0.05, **P < 0.01, ***P < 0.001

We further explored the role of *BCL2A1* in regulating HCT-8 cell apoptosis and parasite burden following *C. parvum* infection using the overexpression vector (pcDNA3.1-*BCL2A1*) or small interfering RNA (siRNA) (si-*BCL2A1*) to upregulate or knock down *BCL2A1* expression, respectively, in HCT-8 cells (Fig. 3a). Similar to the results of inhibiting miR-3976, transfection with pcDNA3.1-*BCL2A1* reduced the percentage of apoptotic cells, while knockdown of *BCL2A1* with siRNA increased the apoptosis rate of HCT-8 cells after infection (Fig. 3b). No changes in parasite burden were detected among the different groups at 2 hpi (Fig. 3c); however the parasite burden was significantly increased at 48 hpi in cells transfected with pcDNA3.1-*BCL2A1* compared with pcDNA3.1-NC, and the opposite result was detected in cells transfected with si-*BCL2A1* (Fig. 3c).

To further determine whether miR-3976 targeted *BCL2A1* to regulate HCT-8 cell apoptosis and parasite burden following *C. parvum* infection, we transfected HCT-8 cells with miR-3976 mimics and pcDNA3.1-*BCL2A1* simultaneously. RT-qPCR showed that miR-3976 was markedly increased in cells co-transfected with miR-3976 mimics and pcDNA3.1-NC, compared with cells co-transfected with miR-3976 mimic NC and pcDNA3.1-NC; however, the increased expression of miR-3976 was eliminated when the cells were co-transfected with miR-3976 mimics and pcDNA3.1-*BCL2A1*, suggesting that miR-3976 directly targeted *BCL2A1* (Fig. 3d). Similarly, flow cytometry showed that the increased apoptosis rate associated with miR-3976 expression was partially countered by overexpressed *BCL2A1* (Fig. 3e), indicating that miR-3976 targeted *BCL2A1* to regulate HCT-8 cell apoptosis following *C. parvum* infection. No changes in parasite burden were detected among the different groups at 2 hpi (Fig. 3f). Furthermore, co-transfection showed that the low parasite burden associated with miR-3976 expression in HCT-8 cells was significantly increased by pcDNA3.1-*BCL2A1* at 48 hpi (Fig. 3f), suggesting that miR-3976 inhibited the *C. parvum* burden in HCT-8 cells by targeting *BCL2A1*.

## Discussion

MiRNAs have been demonstrated to be involved in regulating the innate immune response to *C. parvum* infection [28]. The host’s innate immune response restricts parasite growth and initiates the adaptive immune response, which is necessary for parasite clearance and recovery [29]. Previous studies using an in vitro model of human biliary cryptosporidiosis showed that functional inhibition of nuclear factor kappa-light-chain-enhancer of activated B cells (*NF-κB*) p65-dependent miRNAs (miR-125b, miR-23b, and miR-30b) in cholangiocytes increased the *C. parvum* burden [25], but the underlying mechanisms remained unclear. Inhibition of miR-27b in host cells increased the *C. parvum* infection burden and regulated the epithelial defense against *C. parvum* infection in vitro [20]. In addition, the epithelial defense response against *C. parvum* was increased by upregulation of toll-like receptor 4 (*TLR4*) expression regulated by *let-7i*, and *let-7i* and TLR4 expression regulated the parasite burden and were involved in cholangiocyte immune responses against *C. parvum* infection in vitro [19]. In the current study, miR-3976 expression was induced by *C. parvum* infection and miR-3976 inhibited the *C. parvum* burden in HCT-8 cells by targeting *BCL2A1*, suggesting that this interaction may be involved in the host immune response against *C. parvum* infection by facilitating parasite clearance.

*Cryptosporidium parvum* has developed strategies of immune evasion to counteract the host defense response. Host cell apoptosis following invasion is a common antimicrobial strategy [30]. Previous studies suggested that *C. parvum* infection inhibited intestinal epithelial cell apoptosis during the early stage of infection and promoted host cell apoptosis at the late-infection stage [31]. *B7-H1* (also known as programmed death-ligand 1) was induced by miR-513, thus contributing to the regulation of infected epithelial cell apoptosis and triggering apoptotic cell death by activating the Fas/FasL pathway or programmed cell death protein 1 signaling in bystander cells, including uninfected epithelial cells and infiltrating immune cells [17, 18]. The mechanism of apoptosis/anti-apoptosis may modulate parasite propagation and survival [16, 31]. In our study, miR-3976 targeted *BCL2A1* to modulate HCT-8 cell apoptosis following *C. parvum* infection and miR-3976 also inhibited the *C. parvum* burden in HCT-8 cells by targeting *BCL2A1.* These results suggested that miR-3976 targeting of *BCL2A1* may modulate the parasite burden in HCT-8 cells by regulating cell apoptosis. Further studies using a separation culture system of infected and uninfected cells should be carried out to clarify the relationship between cell apoptosis and parasite burden in infected cells.

*BCL2A1* is a *Bcl-2* anti-apoptotic protein involved in cell apoptosis, autophagy, inflammation, and other biological functions [32–35]. *BCL2A1* transcription is highly regulated. *BCL2A1* is an *NF-κB* target gene with an important role in the innate immune response. *BCL2A1* expression was shown to depend on *NF-κB* activity, and can substitute for *NF-κB* to suppress tumor necrosis factor-α-induced apoptosis [36]. Previous studies suggested that activation of *NF-κB* inhibited infected epithelial cell apoptosis and facilitated parasite survival [37]. Activation of the TLR/NF-ĸB signaling pathway in host cells may trigger the transcriptional regulation of miRNA genes [38, 39]. However, it is unclear whether the *NF-κB* signaling pathway can regulate the expression of miR-3976 and whether *NF-κB* is involved in miR-3976 targeting of *BCL2A1* to regulate cell apoptosis.

## Conclusions

In conclusion, our results suggested that miR-3976 and *BCL2A1* expression were induced by *C. parvum* infection, and that miR-3976 targeting of *BCL2A1* may modulate the parasite burden in HCT-8 cells by regulating cell apoptosis in response to *C. parvum* infection. Future studies should determine the mechanism by which *C. parvum* infection regulates miR-3976 expression and the role of miR-3976 in host anti-*C. parvum* immunity in vivo.

## Supplementary Information

**Additional file 1: Table S1.** Primers used in RT-qPCR and sequences used to generate constructs.

**Additional file 2: Table S2.** RNA oligonucleotides for miRNAs and siRNAs.

**Additional file 3: Table S3.** The inserted sequence of *BCL2A1*-WT and *BCL2A1* MUT.

**Additional file 4: Figure S1.** The overexpression plasmid map of *BCL2A1*.

**Additional file 5.** The sequences of pmirGLO-*BCL2A1*-WT and pmirGLO-*BCL2A1*-MUT.

## Data Availability

All remaining data discussed in this report are found in the main figures or the supplemental materials.

## Abbreviations

*C. parvum*: *Cryptosporidium parvum*
HCT-8 cells: Human ileocecal adenocarcinoma cells
3′-UTR: 3′-Untranslated region
KSRP: KH-type splicing regulatory protein
Fas: Factor-associated suicide
FasL: Fas ligand
RT-qPCR: Real-time quantitative polymerase chain reaction
*BCL2A1*: B-cell lymphoma 2-related protein A1
*NF-κB*: Nuclear factor kappa-light-chain-enhancer of activated B cells
