# Supplementary material for: MiR-3976 regulates HCT-8 cell apoptosis and parasite burden by targeting BCL2A1 in response to Cryptosporidium parvum infection
Source: Parasit Vectors. 2023 Jul 6;16:221. doi: 10.1186/s13071-023-05826-w (PMC10324190; doi:10.1186/s13071-023-05826-w)
Supplement: Supplementary file 1 — Additional file 1: Table S1. Primers used in RT-qPCR and sequences used to generate constructs. [file 13071_2023_5826_MOESM1_ESM.docx]

**Table S1.** Primers used in RT-qPCR and sequences used to generate constructs

| **Target** | **Primers** |  |
| --- | --- | --- |
|  | **Forward** | **Reverse** |
| Human-SSU rRNA | TAG AGA TTG GAG GTT GTT CCT | CTC CAC CAA CTA AGA ACG GCC |
| *C. parvum-*SSU rRNA | CCG ATA ACG AAC GAG ACT CTG G | TAG GGT AGG CAC ACG CTG AGC C |
| BCL2A1 | AAA TTG CCC CGG ATG TGG AT | ACA AAG CCA TTT TCC CAG CCT |
| TRAIL | TGC GTG CTG ATC GTG ATC TTC | GCT CGT TGG TAA AGT ACA CGT A |
| GAPDH | GTC AGC CGC ATC TTC TTT TG | GCG CCC AAT ACG ACC AAA TC |
| U6 | TGG AAC GCT TCA CGA ATT TGC G | GGA ACG ATA CAG AGA AGA TTA GC |
