# Supplementary material for: MiR-3976 regulates HCT-8 cell apoptosis and parasite burden by targeting BCL2A1 in response to Cryptosporidium parvum infection
Source: Parasit Vectors. 2023 Jul 6;16:221. doi: 10.1186/s13071-023-05826-w (PMC10324190; doi:10.1186/s13071-023-05826-w)
Supplement: Supplementary file 2 — Additional file 2: Table S2. RNA oligonucleotides for miRNAs and siRNAs. [file 13071_2023_5826_MOESM2_ESM.docx]

**Table S2.** RNA oligonucleotides for miRNAs and siRNAs

| **Target** | **Sense** | **Antisense** | |
| --- | --- | --- | --- |
| miR-3976 mimics | UAU AGA GAG CAG GAA GAU UAA UGU | | AUU AAU CUU CCU GCU CUC UCU AUA UU |
| miR-3976 mimics NC | UUC UCC GAA CGU GUC ACG UTT | | ACG UGA CAC GUU CGG AGA ATT |
| miR-3976 inhibitor | ACA UUA AUC UUC CUG CUC UCU AUA | |  |
| miR-3976 inhibitor NC | CAG UAC UUU UGU GUA GUA CAA | |  |
| si-BCL2A1 | UGC GUC CUA CAG AUA CCA CAA TT | | UUG UGG UAU CUG UAG GAC GCA TT |
| si- BCL2A1-NC | UUC UCC GAA CGU GUC ACG UTT | | ACG UGA CAC GUU CGG AGA ATT |
