# Supplementary material for: MiR-3976 regulates HCT-8 cell apoptosis and parasite burden by targeting BCL2A1 in response to Cryptosporidium parvum infection
Source: Parasit Vectors. 2023 Jul 6;16:221. doi: 10.1186/s13071-023-05826-w (PMC10324190; doi:10.1186/s13071-023-05826-w)
Supplement: Supplementary file 3 — Additional file 3: Table S3. The inserted sequence of BCL2A1-WT and BCL2A1 MUT. [file 13071_2023_5826_MOESM3_ESM.docx]

**Table S3.** The inserted sequence of BCL2A1-WT and BCL2A1 MUT

| **Target** | **sense** |
| --- | --- |
| BCL2A1-WT | AATTGTATGTATTTTTCTCTATAAATTGTATGTATTTTTCTCTATAAATTGTATGTATTTT |
| BCL2A1-MUT | AATTGTATGTATTTTCTGACGAGAATTGTATGTATTTTCTGACGAGAATTGTATGTATTTT |
