# Supplementary figures and images for: MiR-3976 regulates HCT-8 cell apoptosis and parasite burden by targeting BCL2A1 in response to Cryptosporidium parvum infection
Source: Parasit Vectors. 2023 Jul 6;16:221. doi: 10.1186/s13071-023-05826-w (PMC10324190; doi:10.1186/s13071-023-05826-w)

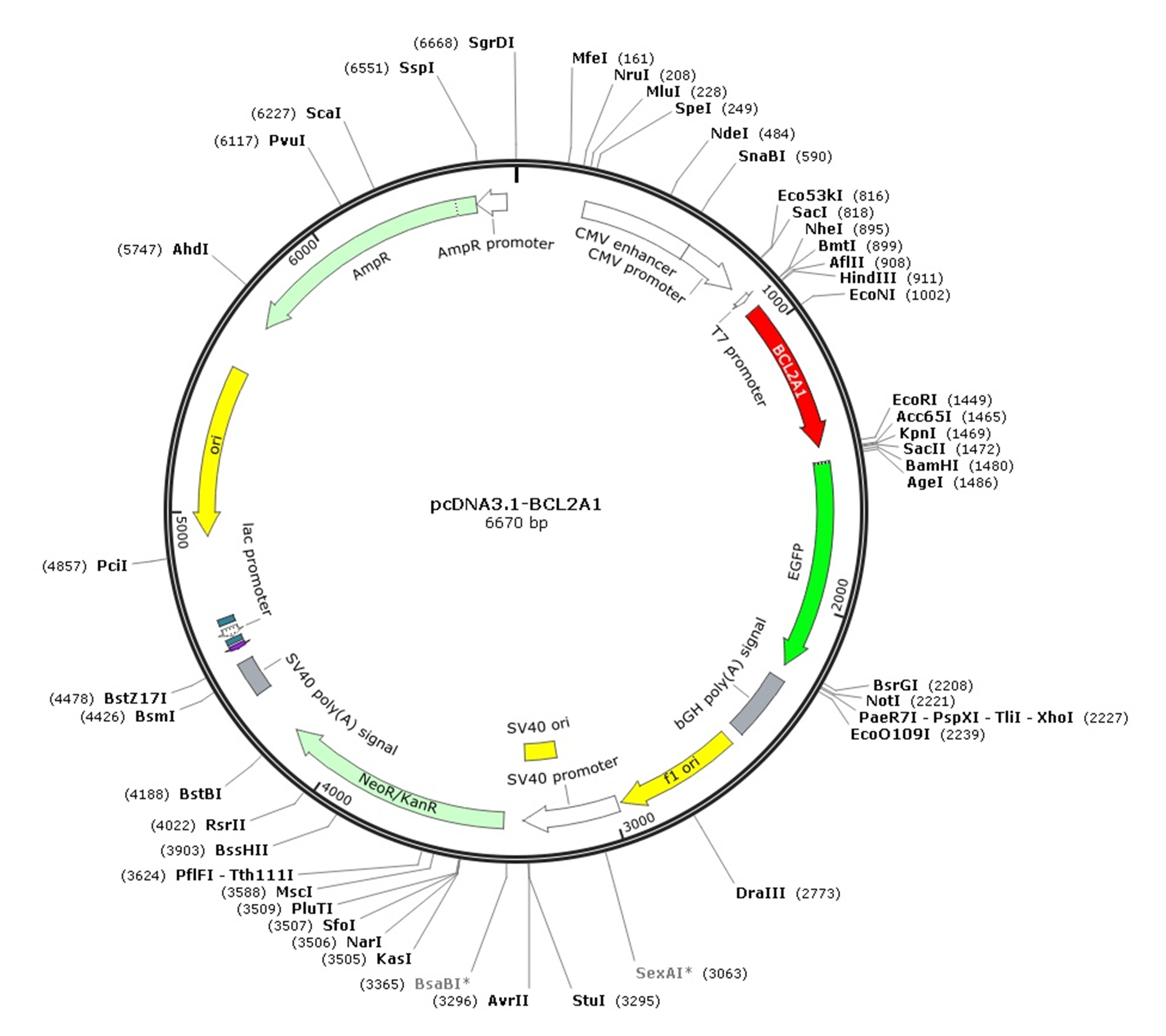

Supplement: Supplementary file 4 — Additional file 4: Figure S1. The overexpression plasmid map of BCL2A1. [file 13071_2023_5826_MOESM4_ESM.tif]
