# Supplementary material for: MiR-3976 regulates HCT-8 cell apoptosis and parasite burden by targeting BCL2A1 in response to Cryptosporidium parvum infection
Source: Parasit Vectors. 2023 Jul 6;16:221. doi: 10.1186/s13071-023-05826-w (PMC10324190; doi:10.1186/s13071-023-05826-w)
Supplement: Supplementary file 5 — Additional file 5. The sequences of pmirGLO-BCL2A1-WT and pmirGLO-BCL2A1-MUT. [file 13071_2023_5826_MOESM5_ESM.docx]

**The sequence of pmirGLO-BCL2A1-WT:**

GAAAGGAAGCTGAGTTGGCTGCTGCCACCGCTGAGCAATAACTAGCATAACCCCTTGGGGCGGCCGCTTCGAGCAGACATGATAAGATACATTGATGAGTTTGGACAAACCACAACTAGAATGCAGTGAAAAAAATGCTTTATTTGTGAAATTTGTGATGCTATTGCTTTATTTGTAACCATTATAAGCTGCAATAAACAAGTTAACAACAACAATTGCATTCATTTTATGTTTCAGGTTCAGGGGGAGATGTGGGAGGTTTTTTTAAGCAAGTAAAACCTCTACAAATGTGGTAAAATCGAATTTTAACAAAATATTAACGCTTACAATTTCCTGATGCGGTATTTTCTCCTTACGCATCTGTGCGGTATTTCACACCGCATACGCGGATCTGCGCAGCACCATGGCCTGAAATAACCTCTGAAAGAGGAACTTGGTTAGGTACCTTCTGAGGCGGAAAGAACCAGCTGTGGAATGTGTGTCAGTTAGGGTGTGGAAAGTCCCCAGGCTCCCCAGCAGGCAGAAGTATGCAAAGCATGCATCTCAATTAGTCAGCAACCAGGTGTGGAAAGTCCCCAGGCTCCCCAGCAGGCAGAAGTATGCAAAGCATGCATCTCAATTAGTCAGCAACCATAGTCCCGCCCCTAACTCCGCCCATCCCGCCCCTAACTCCGCCCAGTTCCGCCCATTCTCCGCCCCATGGCTGACTAATTTTTTTTATTTATGCAGAGGCCGAGGCCGCCTCGGCCTCTGAGCTATTCCAGAAGTAGTGAGGAGGCTTTTTTGGAGGCCTAGGCTTTTGCAAAAAGCTTGATTCTTCTGACACAACAGTCTCGAACCAAAGGCTGGAGCCACCATGGCTTCCAAGGTGTACGACCCCGAGCAACGCAAACGCATGATCACTGGGCCTCAGTGGTGGGCTCGCTGCAAGCAAATGAACGTGCTGGACTCCTTCATCAACTACTATGATTCCGAGAAGCACGCCGAGAACGCCGTGATTTTTCTGCATGGTAACGCTGCCTCCAGCTACCTGTGGAGGCACGTCGTGCCTCACATCGAGCCCGTGGCTAGATGCATCATCCCTGATCTGATCGGAATGGGTAAGTCCGGCAAGAGCGGGAATGGCTCATATCGCCTCCTGGATCACTACAAGTACCTCACCGCTTGGTTCGAGCTGCTGAACCTTCCAAAGAAAATCATCTTTGTGGGCCACGACTGGGGGGCTTGTCTGGCCTTTCACTACTCCTACGAGCACCAAGACAAGATCAAGGCCATCGTCCATGCTGAGAGTGTCGTGGACGTGATCGAGTCCTGGGACGAGTGGCCTGACATCGAGGAGGATATCGCCCTGATCAAGAGCGAAGAGGGCGAGAAAATGGTGCTTGAGAATAACTTCTTCGTCGAGACCATGCTCCCAAGCAAGATCATGCGGAAACTGGAGCCTGAGGAGTTCGCTGCCTACCTGGAGCCATTCAAGGAGAAGGGCGAGGTTAGACGGCCTACCCTCTCCTGGCCTCGCGAGATCCCTCTCGTTAAGGGAGGCAAGCCCGACGTCGTCCAGATTGTCCGCAACTACAACGCCTACCTTCGGGCCAGCGACGATCTGCCTAAGATGTTCATCGAGTCCGACCCTGGGTTCTTTTCCAACGCTATTGTCGAGGGAGCTAAGAAGTTCCCTAACACCGAGTTCGTGAAGGTGAAGGGCCTCCACTTCAGCCAGGAGGACGCTCCAGATGAAATGGGTAAGTACATCAAGAGCTTCGTGGAGCGCGTGCTGAAGAACGAGCAGACCGGTGGTGGGAGCGGAGGTGGCGGATCAGGTGGCGGAGGCTCCGGAGGGATTGAACAAGATGGATTGCACGCAGGTTCTCCGGCCGCTTGGGTGGAGAGGCTATTCGGCTATGACTGGGCACAACAGACAATCGGCTGCTCTGATGCCGCCGTGTTCCGGCTGTCAGCGCAGGGGCGCCCGGTTCTTTTTGTCAAGACCGACCTGTCCGGTGCCCTGAATGAACTGCAGGACGAGGCAGCGCGGCTATCGTGGCTGGCCACGACGGGCGTTCCTTGCGCAGCTGTGCTCGACGTTGTCACTGAAGCGGGAAGGGACTGGCTGCTATTGGGCGAAGTGCCGGGGCAGGATCTCCTGTCATCTCACCTTGCTCCTGCCGAGAAAGTATCCATCATGGCTGATGCAATGCGGCGGCTGCATACGCTTGATCCGGCTACCTGCCCATTCGACCACCAAGCGAAACATCGCATCGAGCGAGCACGTACTCGGATGGAAGCCGGTCTTGTCGATCAGGATGATCTGGACGAAGAGCATCAGGGGCTCGCGCCAGCCGAACTGTTCGCCAGGCTCAAGGCGCGCATGCCCGACGGCGAGGATCTCGTCGTGACCCATGGCGATGCCTGCTTGCCGAATATCATGGTGGAAAATGGCCGCTTTTCTGGATTCATCGACTGTGGCCGGCTGGGTGTGGCGGACCGCTATCAGGACATAGCGTTGGCTACCCGTGATATTGCTGAAGAGCTTGGCGGCGAATGGGCTGACCGCTTCCTCGTGCTTTACGGTATCGCCGCTCCCGATTCGCAGCGCATCGCCTTCTATCGCCTTCTTGACGAGTTCTTCTGAGCGGGACTCTGGGGTTCGAAATGACCGACCAAGCGACGCCCAACCTGCCATCACGATGGCCGCAATAAAATATCTTTATTTTCATTACATCTGTGTGTTGGTTTTTTGTGTGAATCGATAGCGATAAGGATCCTCTTTGCGCTTGCGTTTTCCCTTGTCCAGATAGCCCAGTAGCTGACATTCATCCGGGGTCAGCACCGTTTCTGCGGACTGGCTTTCTACGTAATGGTTTCTTAGACGTCAGGTGGCACTTTTCGGGGAAATGTGCGCGGAACCCCTATTTGTTTATTTTTCTAAATACATTCAAATATGTATCCGCTCATGAGACAATAACCCTGATAAATGCTTCAATAATATTGAAAAAGGAAGAGTATGAGTATTCAACATTTCCGTGTCGCCCTTATTCCCTTTTTTGCGGCATTTTGCCTTCCTGTTTTTGCTCACCCAGAAACGCTGGTGAAAGTAAAAGATGCTGAAGATCAGTTGGGTGCACGAGTGGGTTACATCGAACTGGATCTCAACAGCGGTAAGATCCTTGAGAGTTTTCGCCCCGAAGAACGTTTTCCAATGATGAGCACTTTCAAAGTTCTGCTATGTGGCGCGGTATTATCCCGTATTGACGCCGGGCAAGAGCAACTCGGTCGCCGCATACACTATTCTCAGAATGACTTGGTTGAGTACTCACCAGTCACAGAAAAGCATCTTACGGATGGCATGACAGTAAGAGAATTATGCAGTGCTGCCATAACCATGAGTGATAACACTGCGGCCAACTTACTTCTGACAACTATCGGAGGACCGAAGGAGCTAACCGCTTTTTTGCACAACATGGGGGATCATGTAACTCGCCTTGATCGTTGGGAACCGGAGCTGAATGAAGCCATACCAAACGACGAGCGTGACACCACGATGCCTGTAGCAATGGCAACAACGTTGCGCAAACTATTAACTGGCGAACTACTTACTCTAGCTTCCCGGCAACAATTAATAGACTGGATGGAGGCGGATAAAGTTGCAGGACCACTTCTGCGCTCGGCCCTTCCGGCTGGCTGGTTTATTGCTGATAAATCTGGAGCCGGTGAGCGTGGGTCTCGCGGTATCATTGCAGCACTGGGGCCAGATGGTAAGCCCTCCCGTATCGTAGTTATCTACACGACGGGGAGTCAGGCAACTATGGATGAACGAAATAGACAGATCGCTGAGATAGGTGCCTCACTGATTAAGCATTGGTAATTCGAAATGACCGACCAAGCGACGCCCAACCGGTATCAGCTCACTCAAAGGCGGTAATACGGTTATCCACAGAATCAGGGGATAACGCAGGAAAGAACATGTGAGCAAAAGGCCAGCAAAAGGCCAGGAACCGTAAAAAGGCCGCGTTGCTGGCGTTTTTCCATAGGCTCCGCCCCCCTGACGAGCATCACAAAAATCGACGCTCAAGTCAGAGGTGGCGAAACCCGACAGGACTATAAAGATACCAGGCGTTTCCCCCTGGAAGCTCCCTCGTGCGCTCTCCTGTTCCGACCCTGCCGCTTACCGGATACCTGTCCGCCTTTCTCCCTTCGGGAAGCGTGGCGCTTTCTCATAGCTCACGCTGTAGGTATCTCAGTTCGGTGTAGGTCGTTCGCTCCAAGCTGGGCTGTGTGCACGAACCCCCCGTTCAGCCCGACCGCTGCGCCTTATCCGGTAACTATCGTCTTGAGTCCAACCCGGTAAGACACGACTTATCGCCACTGGCAGCAGCCACTGGTAACAGGATTAGCAGAGCGAGGTATGTAGGCGGTGCTACAGAGTTCTTGAAGTGGTGGCCTAACTACGGCTACACTAGAAGGACAGTATTTGGTATCTGCGCTCTGCTGAAGCCAGTTACCTTCGGAAAAAGAGTTGGTAGCTCTTGATCCGGCAAACAAACCACCGCTGGTAGCGGTGGTTTTTTTGTTTGCAAGCAGCAGATTACGCGCAGAAAAAAAGGATTTCAAGAAGATCCTTTGATCTTTTCTACGGGGTCTGACGCTCAGTGGAACGAAAACTCACGTTAAGGGATTTTGGTCATGAGATTATCAAAAAGGATCTTCACCTAGATCCTTTTATAGTCCGGAAATACAGGAACGCACGCTGGATGGCCCTTCGCTGGGATGGTGAAACCATGAAAAATGGCAGCTTCAGTGGATTAAGTGGGGGTAATGTGGCCTGTACCCTCTGGTTGCATAGGTATTCATACGGTTAAAATTTATCAGGCGCGATTGCGGCAGTTTTTCGGGTGGTTTGTTGCCATTTTTACCTGTCTGCTGCCGTGATCGCGCTGAACGCGTTTTAGCGGTGCGTACAATTAAGGGATTATGGTAAATCCACTTACTGTCTGCCCTCGTAGCCATCGAGATAAACCGCAGTACTCCGGCCACGATGCGTCCGGCGTAGAGGATCGAGATCTACCGGGTAGGGGAGGCGCTTTTCCCAAGGCAGTCTGGAGCATGCGCTTTAGCAGCCCCGCTGGGCACTTGGCGCTACACAAGTGGCCTCTGGCCTCGCACACATTCCACATCCACCGGTAGGCGCCAACCGGCTCCGTTCTTTGGTGGCCCCTTCGCGCCACCTTCTACTCCTCCCCTAGTCAGGAAGTTCCCCCCCGCCCCGCAGCTCGCGTCGTGCAGGACGTGACAAATGGAAGTAGCACGTCTCACTAGTCTCGTGCAGATGGACAGCACCGCTGAGCAATGGAAGCGGGTAGGCCTTTGGGGCAGCGGCCAATAGCAGCTTTGCTCCTTCGCTTTCTGGGCTCAGAGGCTGGGAAGGGGTGGGTCCGGGGGCGGGCTCAGGGGCGGGCTCAGGGGCGGGGCGGGCGCCCGAAGGTCCTCCGGAGGCCCGGCATTCTGCACGCTTCAAAAGCGCACGTCTGCCGCGCTGTTCTCCTCTTCCTCATCTCCGGGCCTTTCGACCTGCAGCCCAAGCTTGGCAATCCGGTACTGTTGGTAAAGCCACCATGGAAGATGCCAAAAACATTAAGAAGGGCCCAGCGCCATTCTACCCACTCGAAGACGGGACCGCCGGCGAGCAGCTGCACAAAGCCATGAAGCGCTACGCCCTGGTGCCCGGCACCATCGCCTTTACCGACGCACATATCGAGGTGGACATTACCTACGCCGAGTACTTCGAGATGAGCGTTCGGCTGGCAGAAGCTATGAAGCGCTATGGGCTGAATACAAACCATCGGATCGTGGTGTGCAGCGAGAATAGCTTGCAGTTCTTCATGCCCGTGTTGGGTGCCCTGTTCATCGGTGTGGCTGTGGCCCCAGCTAACGACATCTACAACGAGCGCGAGCTGCTGAACAGCATGGGCATCAGCCAGCCCACCGTCGTATTCGTGAGCAAGAAAGGGCTGCAAAAGATCCTCAACGTGCAAAAGAAGCTACCGATCATACAAAAGATCATCATCATGGATAGCAAGACCGACTACCAGGGCTTCCAAAGCATGTACACCTTCGTGACTTCCCATTTGCCACCCGGCTTCAACGAGTACGACTTCGTGCCCGAGAGCTTCGACCGGGACAAAACCATCGCCCTGATCATGAACAGTAGTGGCAGTACCGGATTGCCCAAGGGCGTAGCCCTACCGCACCGCACCGCTTGTGTCCGATTCAGTCATGCCCGCGACCCCATCTTCGGCAACCAGATCATCCCCGACACCGCTATCCTCAGCGTGGTGCCATTTCACCACGGCTTCGGCATGTTCACCACGCTGGGCTACTTGATCTGCGGCTTTCGGGTCGTGCTCATGTACCGCTTCGAGGAGGAGCTATTCTTGCGCAGCTTGCAAGACTATAAGATTCAATCTGCCCTGCTGGTGCCCACACTATTTAGCTTCTTCGCTAAGAGCACTCTCATCGACAAGTACGACCTAAGCAACTTGCACGAGATCGCCAGCGGCGGGGCGCCGCTCAGCAAGGAGGTAGGTGAGGCCGTGGCCAAACGCTTCCACCTACCAGGCATCCGCCAGGGCTACGGCCTGACAGAAACAACCAGCGCCATTCTGATCACCCCCGAAGGGGACGACAAGCCTGGCGCAGTAGGCAAGGTGGTGCCCTTCTTCGAGGCTAAGGTGGTGGACTTGGACACCGGTAAGACACTGGGTGTGAACCAGCGCGGCGAGCTGTGCGTCCGTGGCCCCATGATCATGAGCGGCTACGTTAACAACCCCGAGGCTACAAACGCTCTCATCGACAAGGACGGCTGGCTGCACAGCGGCGACATCGCCTACTGGGACGAGGACGAGCACTTCTTCATCGTGGACCGGCTGAAGAGCCTGATCAAATACAAGGGCTACCAGGTAGCCCCAGCCGAACTGGAGAGCATCCTGCTGCAACACCCCAACATCTTCGACGCCGGGGTCGCCGGCCTGCCCGACGACGATGCCGGCGAGCTGCCCGCCGCAGTCGTCGTGCTGGAACACGGTAAAACCATGACCGAGAAGGAGATCGTGGACTATGTGGCCAGCCAGGTTACAACCGCCAAGAAGCTGCGCGGTGGTGTTGTGTTCGTGGACGAGGTGCCTAAAGGACTGACCGGCAAGTTGGACGCCCGCAAGATCCGCGAGATTCTCATTAAGGCCAAGAAGGGCGGCAAGATCGCCGTGTAATTCTAGTTGTTTAAACGAGCTCAATTGTATGTATTTTTCTCTATAAATTGTATGTATTTTTCTCTATAAATTGTATGTATTTTTCTAGAGTCGACCTGCAGGCATGCAAGCTGATCCGGCTGCTAACAAAGCCC

**The sequence of pmirGLO-BCL2A1-MUT:**

GAAAGGAAGCTGAGTTGGCTGCTGCCACCGCTGAGCAATAACTAGCATAACCCCTTGGGGCGGCCGCTTCGAGCAGACATGATAAGATACATTGATGAGTTTGGACAAACCACAACTAGAATGCAGTGAAAAAAATGCTTTATTTGTGAAATTTGTGATGCTATTGCTTTATTTGTAACCATTATAAGCTGCAATAAACAAGTTAACAACAACAATTGCATTCATTTTATGTTTCAGGTTCAGGGGGAGATGTGGGAGGTTTTTTTAAGCAAGTAAAACCTCTACAAATGTGGTAAAATCGAATTTTAACAAAATATTAACGCTTACAATTTCCTGATGCGGTATTTTCTCCTTACGCATCTGTGCGGTATTTCACACCGCATACGCGGATCTGCGCAGCACCATGGCCTGAAATAACCTCTGAAAGAGGAACTTGGTTAGGTACCTTCTGAGGCGGAAAGAACCAGCTGTGGAATGTGTGTCAGTTAGGGTGTGGAAAGTCCCCAGGCTCCCCAGCAGGCAGAAGTATGCAAAGCATGCATCTCAATTAGTCAGCAACCAGGTGTGGAAAGTCCCCAGGCTCCCCAGCAGGCAGAAGTATGCAAAGCATGCATCTCAATTAGTCAGCAACCATAGTCCCGCCCCTAACTCCGCCCATCCCGCCCCTAACTCCGCCCAGTTCCGCCCATTCTCCGCCCCATGGCTGACTAATTTTTTTTATTTATGCAGAGGCCGAGGCCGCCTCGGCCTCTGAGCTATTCCAGAAGTAGTGAGGAGGCTTTTTTGGAGGCCTAGGCTTTTGCAAAAAGCTTGATTCTTCTGACACAACAGTCTCGAACCAAAGGCTGGAGCCACCATGGCTTCCAAGGTGTACGACCCCGAGCAACGCAAACGCATGATCACTGGGCCTCAGTGGTGGGCTCGCTGCAAGCAAATGAACGTGCTGGACTCCTTCATCAACTACTATGATTCCGAGAAGCACGCCGAGAACGCCGTGATTTTTCTGCATGGTAACGCTGCCTCCAGCTACCTGTGGAGGCACGTCGTGCCTCACATCGAGCCCGTGGCTAGATGCATCATCCCTGATCTGATCGGAATGGGTAAGTCCGGCAAGAGCGGGAATGGCTCATATCGCCTCCTGGATCACTACAAGTACCTCACCGCTTGGTTCGAGCTGCTGAACCTTCCAAAGAAAATCATCTTTGTGGGCCACGACTGGGGGGCTTGTCTGGCCTTTCACTACTCCTACGAGCACCAAGACAAGATCAAGGCCATCGTCCATGCTGAGAGTGTCGTGGACGTGATCGAGTCCTGGGACGAGTGGCCTGACATCGAGGAGGATATCGCCCTGATCAAGAGCGAAGAGGGCGAGAAAATGGTGCTTGAGAATAACTTCTTCGTCGAGACCATGCTCCCAAGCAAGATCATGCGGAAACTGGAGCCTGAGGAGTTCGCTGCCTACCTGGAGCCATTCAAGGAGAAGGGCGAGGTTAGACGGCCTACCCTCTCCTGGCCTCGCGAGATCCCTCTCGTTAAGGGAGGCAAGCCCGACGTCGTCCAGATTGTCCGCAACTACAACGCCTACCTTCGGGCCAGCGACGATCTGCCTAAGATGTTCATCGAGTCCGACCCTGGGTTCTTTTCCAACGCTATTGTCGAGGGAGCTAAGAAGTTCCCTAACACCGAGTTCGTGAAGGTGAAGGGCCTCCACTTCAGCCAGGAGGACGCTCCAGATGAAATGGGTAAGTACATCAAGAGCTTCGTGGAGCGCGTGCTGAAGAACGAGCAGACCGGTGGTGGGAGCGGAGGTGGCGGATCAGGTGGCGGAGGCTCCGGAGGGATTGAACAAGATGGATTGCACGCAGGTTCTCCGGCCGCTTGGGTGGAGAGGCTATTCGGCTATGACTGGGCACAACAGACAATCGGCTGCTCTGATGCCGCCGTGTTCCGGCTGTCAGCGCAGGGGCGCCCGGTTCTTTTTGTCAAGACCGACCTGTCCGGTGCCCTGAATGAACTGCAGGACGAGGCAGCGCGGCTATCGTGGCTGGCCACGACGGGCGTTCCTTGCGCAGCTGTGCTCGACGTTGTCACTGAAGCGGGAAGGGACTGGCTGCTATTGGGCGAAGTGCCGGGGCAGGATCTCCTGTCATCTCACCTTGCTCCTGCCGAGAAAGTATCCATCATGGCTGATGCAATGCGGCGGCTGCATACGCTTGATCCGGCTACCTGCCCATTCGACCACCAAGCGAAACATCGCATCGAGCGAGCACGTACTCGGATGGAAGCCGGTCTTGTCGATCAGGATGATCTGGACGAAGAGCATCAGGGGCTCGCGCCAGCCGAACTGTTCGCCAGGCTCAAGGCGCGCATGCCCGACGGCGAGGATCTCGTCGTGACCCATGGCGATGCCTGCTTGCCGAATATCATGGTGGAAAATGGCCGCTTTTCTGGATTCATCGACTGTGGCCGGCTGGGTGTGGCGGACCGCTATCAGGACATAGCGTTGGCTACCCGTGATATTGCTGAAGAGCTTGGCGGCGAATGGGCTGACCGCTTCCTCGTGCTTTACGGTATCGCCGCTCCCGATTCGCAGCGCATCGCCTTCTATCGCCTTCTTGACGAGTTCTTCTGAGCGGGACTCTGGGGTTCGAAATGACCGACCAAGCGACGCCCAACCTGCCATCACGATGGCCGCAATAAAATATCTTTATTTTCATTACATCTGTGTGTTGGTTTTTTGTGTGAATCGATAGCGATAAGGATCCTCTTTGCGCTTGCGTTTTCCCTTGTCCAGATAGCCCAGTAGCTGACATTCATCCGGGGTCAGCACCGTTTCTGCGGACTGGCTTTCTACGTAATGGTTTCTTAGACGTCAGGTGGCACTTTTCGGGGAAATGTGCGCGGAACCCCTATTTGTTTATTTTTCTAAATACATTCAAATATGTATCCGCTCATGAGACAATAACCCTGATAAATGCTTCAATAATATTGAAAAAGGAAGAGTATGAGTATTCAACATTTCCGTGTCGCCCTTATTCCCTTTTTTGCGGCATTTTGCCTTCCTGTTTTTGCTCACCCAGAAACGCTGGTGAAAGTAAAAGATGCTGAAGATCAGTTGGGTGCACGAGTGGGTTACATCGAACTGGATCTCAACAGCGGTAAGATCCTTGAGAGTTTTCGCCCCGAAGAACGTTTTCCAATGATGAGCACTTTCAAAGTTCTGCTATGTGGCGCGGTATTATCCCGTATTGACGCCGGGCAAGAGCAACTCGGTCGCCGCATACACTATTCTCAGAATGACTTGGTTGAGTACTCACCAGTCACAGAAAAGCATCTTACGGATGGCATGACAGTAAGAGAATTATGCAGTGCTGCCATAACCATGAGTGATAACACTGCGGCCAACTTACTTCTGACAACTATCGGAGGACCGAAGGAGCTAACCGCTTTTTTGCACAACATGGGGGATCATGTAACTCGCCTTGATCGTTGGGAACCGGAGCTGAATGAAGCCATACCAAACGACGAGCGTGACACCACGATGCCTGTAGCAATGGCAACAACGTTGCGCAAACTATTAACTGGCGAACTACTTACTCTAGCTTCCCGGCAACAATTAATAGACTGGATGGAGGCGGATAAAGTTGCAGGACCACTTCTGCGCTCGGCCCTTCCGGCTGGCTGGTTTATTGCTGATAAATCTGGAGCCGGTGAGCGTGGGTCTCGCGGTATCATTGCAGCACTGGGGCCAGATGGTAAGCCCTCCCGTATCGTAGTTATCTACACGACGGGGAGTCAGGCAACTATGGATGAACGAAATAGACAGATCGCTGAGATAGGTGCCTCACTGATTAAGCATTGGTAATTCGAAATGACCGACCAAGCGACGCCCAACCGGTATCAGCTCACTCAAAGGCGGTAATACGGTTATCCACAGAATCAGGGGATAACGCAGGAAAGAACATGTGAGCAAAAGGCCAGCAAAAGGCCAGGAACCGTAAAAAGGCCGCGTTGCTGGCGTTTTTCCATAGGCTCCGCCCCCCTGACGAGCATCACAAAAATCGACGCTCAAGTCAGAGGTGGCGAAACCCGACAGGACTATAAAGATACCAGGCGTTTCCCCCTGGAAGCTCCCTCGTGCGCTCTCCTGTTCCGACCCTGCCGCTTACCGGATACCTGTCCGCCTTTCTCCCTTCGGGAAGCGTGGCGCTTTCTCATAGCTCACGCTGTAGGTATCTCAGTTCGGTGTAGGTCGTTCGCTCCAAGCTGGGCTGTGTGCACGAACCCCCCGTTCAGCCCGACCGCTGCGCCTTATCCGGTAACTATCGTCTTGAGTCCAACCCGGTAAGACACGACTTATCGCCACTGGCAGCAGCCACTGGTAACAGGATTAGCAGAGCGAGGTATGTAGGCGGTGCTACAGAGTTCTTGAAGTGGTGGCCTAACTACGGCTACACTAGAAGGACAGTATTTGGTATCTGCGCTCTGCTGAAGCCAGTTACCTTCGGAAAAAGAGTTGGTAGCTCTTGATCCGGCAAACAAACCACCGCTGGTAGCGGTGGTTTTTTTGTTTGCAAGCAGCAGATTACGCGCAGAAAAAAAGGATTTCAAGAAGATCCTTTGATCTTTTCTACGGGGTCTGACGCTCAGTGGAACGAAAACTCACGTTAAGGGATTTTGGTCATGAGATTATCAAAAAGGATCTTCACCTAGATCCTTTTATAGTCCGGAAATACAGGAACGCACGCTGGATGGCCCTTCGCTGGGATGGTGAAACCATGAAAAATGGCAGCTTCAGTGGATTAAGTGGGGGTAATGTGGCCTGTACCCTCTGGTTGCATAGGTATTCATACGGTTAAAATTTATCAGGCGCGATTGCGGCAGTTTTTCGGGTGGTTTGTTGCCATTTTTACCTGTCTGCTGCCGTGATCGCGCTGAACGCGTTTTAGCGGTGCGTACAATTAAGGGATTATGGTAAATCCACTTACTGTCTGCCCTCGTAGCCATCGAGATAAACCGCAGTACTCCGGCCACGATGCGTCCGGCGTAGAGGATCGAGATCTACCGGGTAGGGGAGGCGCTTTTCCCAAGGCAGTCTGGAGCATGCGCTTTAGCAGCCCCGCTGGGCACTTGGCGCTACACAAGTGGCCTCTGGCCTCGCACACATTCCACATCCACCGGTAGGCGCCAACCGGCTCCGTTCTTTGGTGGCCCCTTCGCGCCACCTTCTACTCCTCCCCTAGTCAGGAAGTTCCCCCCCGCCCCGCAGCTCGCGTCGTGCAGGACGTGACAAATGGAAGTAGCACGTCTCACTAGTCTCGTGCAGATGGACAGCACCGCTGAGCAATGGAAGCGGGTAGGCCTTTGGGGCAGCGGCCAATAGCAGCTTTGCTCCTTCGCTTTCTGGGCTCAGAGGCTGGGAAGGGGTGGGTCCGGGGGCGGGCTCAGGGGCGGGCTCAGGGGCGGGGCGGGCGCCCGAAGGTCCTCCGGAGGCCCGGCATTCTGCACGCTTCAAAAGCGCACGTCTGCCGCGCTGTTCTCCTCTTCCTCATCTCCGGGCCTTTCGACCTGCAGCCCAAGCTTGGCAATCCGGTACTGTTGGTAAAGCCACCATGGAAGATGCCAAAAACATTAAGAAGGGCCCAGCGCCATTCTACCCACTCGAAGACGGGACCGCCGGCGAGCAGCTGCACAAAGCCATGAAGCGCTACGCCCTGGTGCCCGGCACCATCGCCTTTACCGACGCACATATCGAGGTGGACATTACCTACGCCGAGTACTTCGAGATGAGCGTTCGGCTGGCAGAAGCTATGAAGCGCTATGGGCTGAATACAAACCATCGGATCGTGGTGTGCAGCGAGAATAGCTTGCAGTTCTTCATGCCCGTGTTGGGTGCCCTGTTCATCGGTGTGGCTGTGGCCCCAGCTAACGACATCTACAACGAGCGCGAGCTGCTGAACAGCATGGGCATCAGCCAGCCCACCGTCGTATTCGTGAGCAAGAAAGGGCTGCAAAAGATCCTCAACGTGCAAAAGAAGCTACCGATCATACAAAAGATCATCATCATGGATAGCAAGACCGACTACCAGGGCTTCCAAAGCATGTACACCTTCGTGACTTCCCATTTGCCACCCGGCTTCAACGAGTACGACTTCGTGCCCGAGAGCTTCGACCGGGACAAAACCATCGCCCTGATCATGAACAGTAGTGGCAGTACCGGATTGCCCAAGGGCGTAGCCCTACCGCACCGCACCGCTTGTGTCCGATTCAGTCATGCCCGCGACCCCATCTTCGGCAACCAGATCATCCCCGACACCGCTATCCTCAGCGTGGTGCCATTTCACCACGGCTTCGGCATGTTCACCACGCTGGGCTACTTGATCTGCGGCTTTCGGGTCGTGCTCATGTACCGCTTCGAGGAGGAGCTATTCTTGCGCAGCTTGCAAGACTATAAGATTCAATCTGCCCTGCTGGTGCCCACACTATTTAGCTTCTTCGCTAAGAGCACTCTCATCGACAAGTACGACCTAAGCAACTTGCACGAGATCGCCAGCGGCGGGGCGCCGCTCAGCAAGGAGGTAGGTGAGGCCGTGGCCAAACGCTTCCACCTACCAGGCATCCGCCAGGGCTACGGCCTGACAGAAACAACCAGCGCCATTCTGATCACCCCCGAAGGGGACGACAAGCCTGGCGCAGTAGGCAAGGTGGTGCCCTTCTTCGAGGCTAAGGTGGTGGACTTGGACACCGGTAAGACACTGGGTGTGAACCAGCGCGGCGAGCTGTGCGTCCGTGGCCCCATGATCATGAGCGGCTACGTTAACAACCCCGAGGCTACAAACGCTCTCATCGACAAGGACGGCTGGCTGCACAGCGGCGACATCGCCTACTGGGACGAGGACGAGCACTTCTTCATCGTGGACCGGCTGAAGAGCCTGATCAAATACAAGGGCTACCAGGTAGCCCCAGCCGAACTGGAGAGCATCCTGCTGCAACACCCCAACATCTTCGACGCCGGGGTCGCCGGCCTGCCCGACGACGATGCCGGCGAGCTGCCCGCCGCAGTCGTCGTGCTGGAACACGGTAAAACCATGACCGAGAAGGAGATCGTGGACTATGTGGCCAGCCAGGTTACAACCGCCAAGAAGCTGCGCGGTGGTGTTGTGTTCGTGGACGAGGTGCCTAAAGGACTGACCGGCAAGTTGGACGCCCGCAAGATCCGCGAGATTCTCATTAAGGCCAAGAAGGGCGGCAAGATCGCCGTGTAATTCTAGTTGTTTAAACGAGCTCAATTGTATGTATTTTCTGACGAGAATTGTATGTATTTTCTGACGAGAATTGTATGTATTTTTCTAGAGTCGACCTGCAGGCATGCAAGCTGATCCGGCTGCTAACAAAGCCC
